# Seizures Triggered by Systemic Administration of 4-Aminopyridine in Rats Lead to Acute Brain Glucose Hypometabolism, as Assessed by [^18^F]FDG PET Neuroimaging

**DOI:** 10.3390/ijms252312774

**Published:** 2024-11-28

**Authors:** Francisca Gómez-Oliver, Rubén Fernández de la Rosa, Mirjam Brackhan, Pablo Bascuñana, Miguel Ángel Pozo, Luis García-García

**Affiliations:** 1 Unidad de Cartografía Cerebral, Instituto Pluridisciplinar, Universidad Complutense de Madrid, 28040 Madrid, Spain; gomezof@ucm.es (F.G.-O.); rufernan@ucm.es (R.F.d.l.R.); brackhanm@gmail.com (M.B.); pablo.bascunana@salud.madrid.org (P.B.); pozo@ucm.es (M.Á.P.); 2 Departamento de Farmacología, Farmacognosia y Botánica, Facultad de Farmacia, Universidad Complutense de Madrid, 28040 Madrid, Spain; 3 Instituto de Investigación Sanitaria San Carlos (IdISSC), Hospital Clínico San Carlos, 28040 Madrid, Spain; 4 Infraestructura Científico-Técnica Singular Bioimagen Complutense (ICTS BIOIMAC), Universidad Complutense de Madrid, 28040 Madrid, Spain; 5 Departamento de Fisiología, Facultad de Medicina, Universidad Complutense de Madrid, 28040 Madrid, Spain

**Keywords:** 4-aminopyridine (4-AP), seizures, 2-deoxy-2-[^18^F]fluoro-D-glucose ([^18^F]FDG), positron emission tomography (PET), hypometabolism, neuroinflammation, statistical parametric mapping (SPM), hippocampus, cerebellum

## Abstract

4-aminopyridine (4-AP) is a non-selective blocker of voltage-dependent K^+^ channels used to improve walking in multiple sclerosis patients, and it may be useful in the treatment of cerebellar diseases. In animal models, 4-AP is used as a convulsant agent. When administered intrahippocampally, 4-AP induces acute local glucose hypermetabolism and significant brain damage, while i.p. administration causes less neuronal damage. This study aimed to investigate the effects of a single i.p. administration of 4-AP on acute brain glucose metabolism as well as on neuronal viability and signs of neuroinflammation 3 days after the insult. Brain glucose metabolism was evaluated by [^18^F]FDG PET neuroimaging. [^18^F]FDG uptake was analyzed based on volumes of interest (VOIs) as well as by voxel-based (SPM) analyses. The results showed that independently of the type of data analysis used (VOIs or SPM), 4-AP induced acute generalized brain glucose hypometabolism, except in the cerebellum. Furthermore, the SPM analysis normalized by the whole brain uptake revealed a significant cerebellar hypermetabolism. The neurohistochemical assays showed that 4-AP induced hippocampal astrocyte reactivity 3 days after the insult, without inducing changes in neuronal integrity or microglia-mediated neuroinflammation. Thus, acute brain glucose metabolic and neuroinflammatory profiles in response to i.p. 4-AP clearly differed from that reported for intrahippocampal administration. Finally, the results suggest that the cerebellum might be more resilient to the 4-AP-induced hypometabolism.

## 1. Introduction

4-aminopyridine (4-AP; fampridine) is a non-selective blocker of voltage-dependent K^+^ channels (K_v_) widely used in basic research as a convulsant agent increasing the action potential firing and neurotransmission that leads to acute epileptiform activity [1,2,3,4]. 

Interestingly, in the clinical scenario, the depolarizing action endows 4-AP with the therapeutic effectiveness to improve walking in patients with multiple sclerosis when used at low doses [1,5]. In addition, clinical trials and experimental studies suggest that 4-AP might be useful for the treatment of nystagmus, vertigo, and various other cerebellar disorders [6]. 

The depolarizing action of 4-AP stimulates neurotransmitter release and facilitates inward Ca^2+^ currents, therefore causing neuronal hyperactivity [2,7,8,9,10,11]. Increased excitatory glutamate-mediated neurotransmission in multiple brain areas such as the hippocampus, entorhinal cortex, and striatum is one of the main contributors to the epileptiform activity induced by 4-AP [12,13,14]. This is supported by the protective effects of antagonists of N-methyl-D-aspartate (NMDA) and α-amino-3-hydroxy-5-methyl-4-isoxazolepropionic acid (AMPA) receptors, but not by GABAergic drugs, against 4-AP-induced epileptiform activity [15,16,17,18]. 

Intrahippocampal, cortical, and intraperitoneal 4-AP administration are known to chemically induce seizure activity in rodents and are used as epilepsy models to study neuroprotection [19,20,21]. Nonetheless, in rodents, some behavioral, electrophysiological, excitotoxic, and neuronal damage profiles differ between both routes of administration [2,17,18,20,21,22,23]. 

4-AP has different affinities for the K_v_ subtypes. At low concentrations, it primarily blocks the K_v_1 family, while at high concentrations, like those that induce convulsions, it blocks a wide range of K_v_ channels in neuronal and glial cells [19,24,25,26]. Therefore, 4-AP-induced effects seem to depend significantly on the route of administration, which in turn determines its concentration in the different brain areas as well as the cell types affected. 

2-deoxy-2-[^18^F]fluoro-D-glucose ([^18^F]FDG) positron emission tomography (PET) neuroimaging is a minimally invasive, valuable tool for studying regional cerebral glucose metabolism, being a reflection of its functionality. Furthermore, clinical and basic molecular neuroimaging studies have shown that the brain damage associated either with epilepsy or with seizures is characteristically associated with brain glucose metabolism alterations [20,27,28,29,30]. In this context, we have previously reported that intrahippocampal 4-AP administration induces acute brain hypermetabolism that, reflecting increased local neuronal activity, leads to short-term detrimental consequences in hippocampal integrity and neuroinflammation [21]. 

In the current study, our aim was to evaluate the consequences of a single i.p. administration of 4-AP on brain glucose metabolism during the acute depolarizing phase by using in vivo [^18^F]FDG PET. Our second objective was to determine the short-term (3 days after seizure) consequences on neuronal viability and signs of neuroinflammation by evaluating different markers of neuronal integrity, brain damage, astrocytic activation, and microglia-mediated neuroinflammation. 

## 2. Results

**Figure 1:**
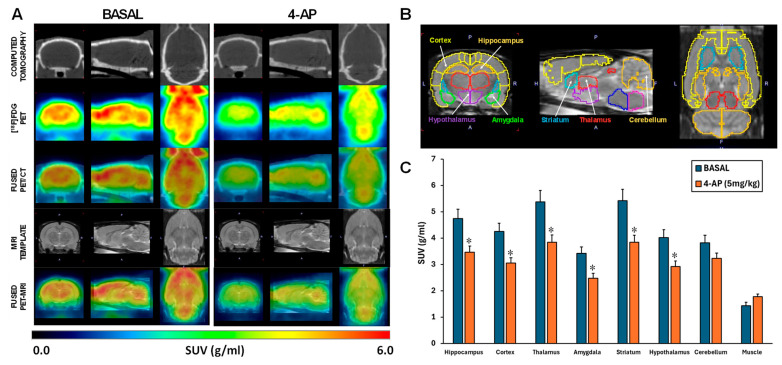
Systemic 4-AP administration (5 mg/kg, i.p.) induced generalized acute brain hypometabolism except in the cerebellum. (**A**). Representative images in coronal, sagittal, and trans-axial views showing [^18^F]FDG uptake under basal and 4-AP treatment conditions. First row: representative CT images; Second row: [^18^F]FDG PET images; Third row: [^18^F]FDG PET/CT fused images; Fourth row: MRI brain template; Fifth row: [^18^F]FDG PET/MRI fused images. (**B**) MRI rat brain template in coronal, sagittal, and transaxial views with the VOIs corresponding to the studied areas. (**C**) [^18^F]FDG uptake under basal and 4-AP treatment conditions is shown as SUV units (mean ± SEM, n = 13 rats/group). Significant differences between 4-AP and BASAL conditions are depicted as: * *p* < 0.01, paired *t*-tests.

The administration of 4-AP was accompanied by intense seizure behavior. In fact, the dose of 5 mg/kg triggered generalized convulsions in all rats between 5 and 10 min after injection, which is in line with our previous work [20]. Additionally, no mortality was recorded at this dose. When the [^18^F]FDG brain uptake was analyzed by SUV, 4-AP induced an acute hypometabolism of approximately 28% in all areas studied (*p* < 0.01) except in the cerebellum (*p* = 0.067) (Figure 1A–C).

**Figure 2:**
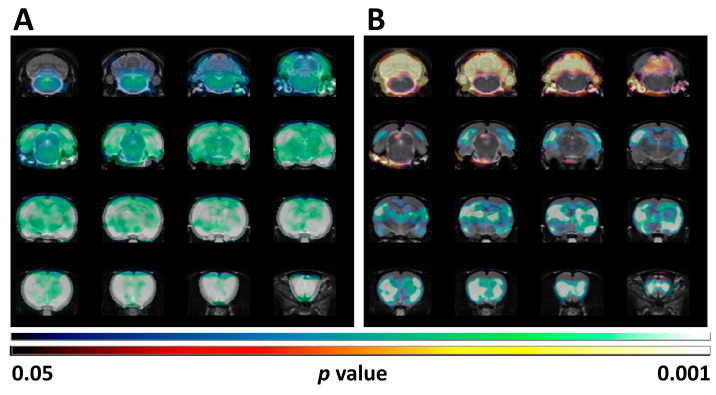
Statistical parametric map (SPM) analysis for FDG-PET metabolic patterns resulting from the comparison between the BASAL and the 4-AP groups is shown overlaid on a rat T2-MRI template in coronal view from olfactory bulb (bottom row) to cerebellum (upper row). (**A**) SPM images obtained after SUV normalization show extensive cerebral hypometabolism (note the lack of effect in most of the cerebellum). (**B**) SPM images obtained after normalization to whole brain [^18^F]FDG uptake showing hypometabolism involving telencephalic and diencephalic structures as well as a statistically significant increase in metabolism in the cerebellum. Upper scale: Color code bar for clusters showing statistically significant hypometabolism (4-AP vs. BASAL); lower scale: Color code bar for clusters showing statistically significant hypermetabolism (4-AP vs. BASAL). Results are shown at *p* < 0.05 (uncorrected for multiple comparisons) with a minimum cluster size of 100 voxels.

These results coincided with those obtained when the SUV differences were analyzed by SPM (Figure 2A). However, when the SPM analysis was performed after normalization of the [^18^F]FDG uptake by the whole brain, the reduced uptake in clusters localized in telencephalic and diencephalic regions was maintained, but a statistically significant increase in the glucose metabolism in the cerebellum was obtained (*p* < 0.05; Figure 2B).

Considering the 4-AP was administered systemically, we also evaluated the SUV [^18^F]FDG uptake in the forepaw muscle. The results showed that 4-AP did not significantly increase the muscle glucose metabolism (*p* = 0.057; Figure 1C).

**Figure 3:**
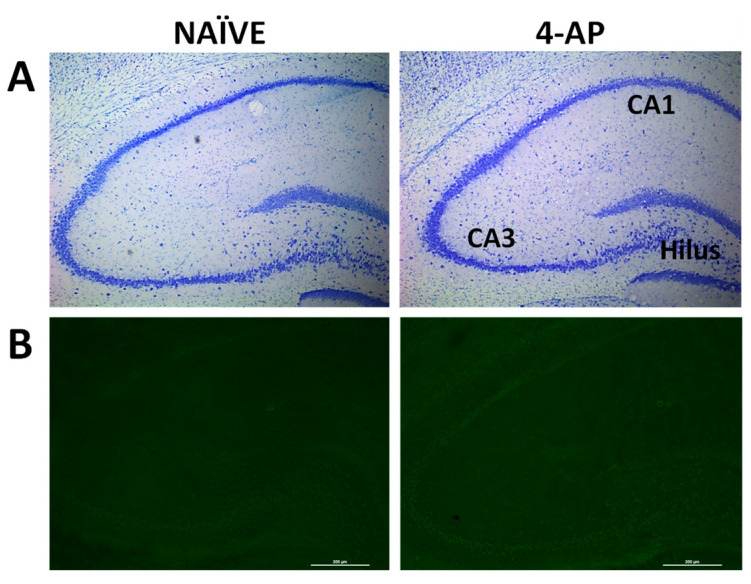
4-AP-induced seizures did not have effects on hippocampal integrity when evaluated 3 days after the insult. (**A**) Representative Nissl (toluidine blue) and (**B**) Fluoro-Jade C fluorescence staining micrographs from NAÏVE and 4-AP-treated rats containing hippocampal CA1, CA3, and dentate gyrus/hilus are shown.

**Figure 4:**
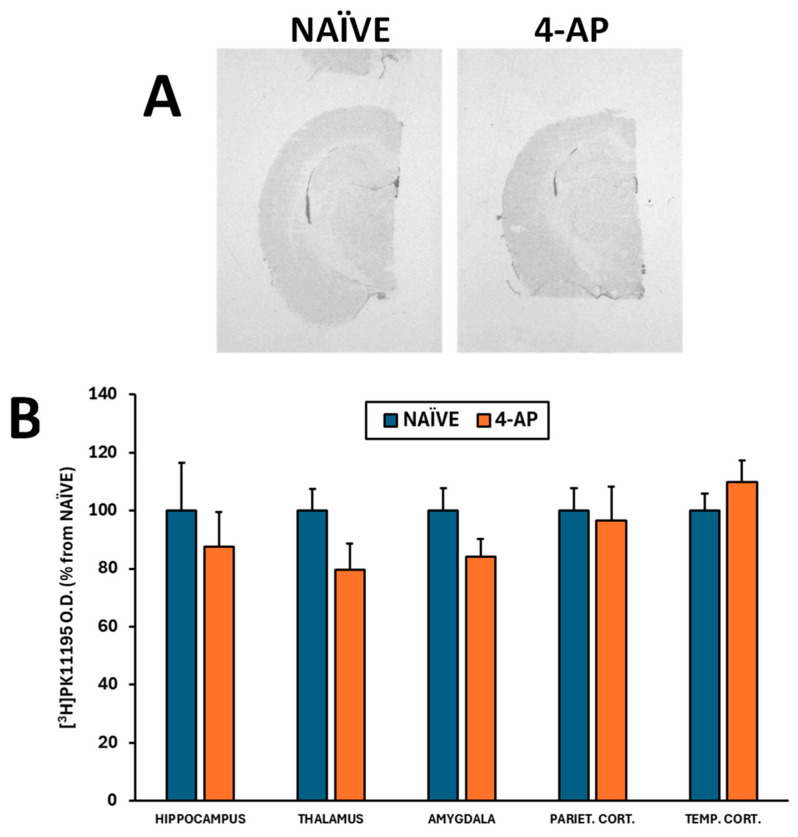
Systemic 4-AP (5 mg/kg, i.p.) administration did not result in signs of microglia-induced neuroinflammation as measured 3 days after the insult by the [^3^H]PK11195 binding in major brain regions involved in convulsant activity. (**A**) Representative [^3^H]PK11195 autoradiograms corresponding to the NAÏVE (n = 6) and 4-AP (n = 13) groups. (**B**) [^3^H]PK11195 binding expressed in O.D. as % from NAÏVE group in various brain regions (not significant; *p* > 0.05 in all areas studied). Data are shown as mean ± SEM.

We also evaluated the effects of 4-AP on neuronal integrity, neurodegeneration, astrocyte reactivity, and microglia-mediated neuroinflammation in the hippocampus 3 days after 4-AP-induced seizures. Our results revealed that 4-AP had no detrimental effects either on hippocampal neurodegeneration (Nissl and Fluoro-Jade C stainings; Figure 3) or on microglia-mediated neuroinflammation (Figure 4A,B; *p* > 0.05 in all areas studied).

**Figure 5:**
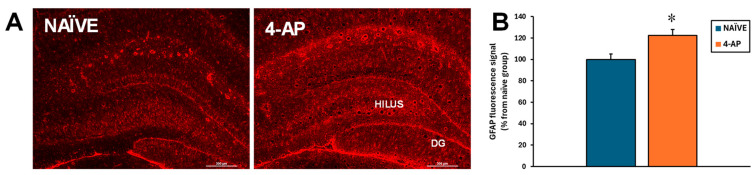
Systemic 4-AP administration (5 mg/kg, i.p.) resulted in hippocampal astrogliosis measured 3 days after the insult. (**A**) Images showing representative GFAP immunofluorescence micrographs at the hilus/dentate gyrus area of NAÏVE (n = 6) and 4-AP-treated rats (n = 13). (**B**) Bar plot corresponding to quantitative data from GFAP immunofluorescence intensity as marker of astrogliosis on the hilus. Data are expressed as percentage of the signal obtained in the NAÏVE group and shown as mean ± SEM. * *p* < 0.05 NAÏVE vs. 4-AP; unpaired *t*-test.

By contrast, 4-AP-induced hippocampal astrocyte reactivity. Thus, the GFAP immunofluorescence showed a significant increase in approximately 22% (*p* < 0.05) of fluorescence signal in the dorsal hippocampus (Figure 5).

## 3. Discussion

In this study we have evaluated brain glucose metabolism by longitudinal [^18^F]FDG PET neuroimaging in rats under baseline conditions as well as, in the same rats, immediately after i.p. 4-AP administration (5 mg/kg). This dose and route of administration resulted in convulsive seizures and acute generalized brain glucose hypometabolism except in the cerebellum. Additionally, 4-AP resulted in astrocyte reactivity measured 3 days after the insult without apparent effects on microglia-mediated neuroinflammation.

### 3.1. VOIs Analysis Showed That 4-AP Administered i.p. Was Followed by Acute Generalized Brain Glucose Hypometabolism 

The current study showed an acute brain hypometabolism in response to 4-AP i.p. injection (Figure 1). This result was at first surprising considering that, on the one hand, all rats responded with a marked convulsive activity, and on the other hand, we had previously reported that, when administered in the hippocampus, 4-AP (7 µg in 5 µL) induced acute local hypermetabolism around the injection site also extending to adjacent ipsilateral areas, particularly temporal and parietal cortices and, to a lesser extent, to contralateral areas [21]. This hypermetabolism is associated with increased local neuronal excitability that results in detrimental consequences in hippocampal integrity and neuroinflammation. Likewise, local hypermetabolism at the intracortical injection site of 4-AP in rats has also been found when measured by the uptake of 2-(*N*-(7-nitrobenz-2-oxa-1,3-diazol-4-yl)amino)-2-deoxyglucose (2-NBDG), a fluorescent deoxyglucose substitute [31].

However, we have also previously shown that systemic 4-AP (3 mg/kg, i.p.) had no effects on brain glucose metabolism, as measured 3 days after the 4-AP administration [20]. Supporting the hypometabolic response described herein, hypometabolism in response to 4-AP-induced seizures has also been reported in mouse hippocampal slices [27]. Thus, it seems the metabolic responses to 4-AP go in the opposite direction depending on the dose and route of administration. Furthermore, when 4-AP is administered systemically, it may induce a dose-dependently transient brain hypometabolism.

Despite these differences, 4-AP is used either by systemic or by intracerebral route as a model to induce seizure activity in rodents [3,20,21]. However, the results highlight that the brain glucose metabolic profile clearly differs between both routes of administration. This route-dependent difference in metabolic profile is in line with the differences reported regarding the differential behavioral, electrophysiologic, as well as neuronal damage profiles that characterize these routes. Thus, i.p. 4-AP administration at doses of 5 mg/kg [22] and 7 mg/kg [2] in adult male rats has been reported to induce generalized tonic convulsions characterized by electrical discharges of relatively short duration, but no extensive neuronal damage is found when administered i.p. at doses of 3 and 5 mg/kg [16,20,22]. By contrast, studies administering 2.1 nmol of 4-AP intrahippocampally [16,22] as well as through microdialysis probes at doses ranging from 0.7 up to 70 mM [17,18] result in limbic seizures and frequent wet-dog shakes that correlate with hippocampal discharges that rapidly propagate to other structures and encompass intense brain damage, including loss of pyramidal neurons. Hippocampal damage has also been reported after intrahippocampal injection of 4-AP (7 μg/5 μL) [21]. The neuronal damage is thought to be linked to glutamate-induced excitotoxicity [17,18] through overactivation of NMDA receptors [32]. And, at low doses, i.c.v. administration of 4-AP produces epileptiform activity without affecting glutamate levels [33]. However, when 4-AP (0.5 mg/kg) was placed on the parietal cortex surface, no widespread cell death has been also reported [23].

In addition to the central excitatory activity, 4-AP is known to have noticeable peripheral effects, increasing synaptic neuromuscular transmission and directly increasing skeletal muscle twitch tension [34,35,36]. It has been suggested that the latter may contribute to their therapeutic effects in multiple sclerosis patients [36]. Considering that in our study 4-AP was administered systemically, and to determine whether the actions of 4-AP on skeletal muscle might have interfered with brain [^18^F]FDG uptake, we measured glucose metabolism in the forepaw muscle. Our results showed that 4-AP did not increase muscle glucose metabolism (Figure 1C), suggesting that the acute brain glucose hypometabolism in response to systemic 4-AP is not due to a higher uptake of [^18^F]FDG by skeletal muscle as a consequence of the convulsive seizures.

### 3.2. Cerebellar Metabolism Is Increased in Response to 4-AP as Evaluated by SPM Normalized to Whole Brain [^18^F]FDG Uptake 

We also evaluated brain glucose metabolism by SPM after normalizing the values to the whole brain [^18^F]FDG uptake. In our current study, this analysis detected significantly hypometabolic clusters that overlapped with the regions detected by traditional VOI analyses. These clusters included telencephalic as well as diencephalic structures (Figure 2). Interestingly, this type of analysis highlighted a significant increase in cerebellar uptake (Figure 2). This type of normalization of regional values *vs.* the whole brain reference value allows detecting the impact of localized changes, minimizes individual differences, and avoids the impact of peripheral uptake.

One limitation associated with the SPM analyses after normalizing the values to the whole brain [^18^F]FDG uptake has been raised when studying diseases characterized by whole brain hypometabolism. It has been noticed that this analysis may lead to underestimation of reduced metabolism in some regions as well as to overestimations in others, which might be inconsistent with the clinical or experimental conditions [37,38,39]. However, in our case, and independently of the type of analysis (VOIs and SPM analyses), our data suggest that the cerebellum might be more resilient to 4-AP-induced hypometabolism. 4-AP has been shown to increase Purkinje cell activity in brain slices of the rat [40,41], and consequently to increase the GABAergic neurotransmission that restores cerebellar inhibition, an effect that has been related to the therapeutic effects of 4-AP on cerebellar disorders [42]. In a clinical case study evaluating brain glucose metabolism by [^18^F]FDG PET, treatment with 4-AP lessened the cerebellar hypometabolism associated with downbeat nystagmus. The authors suggested that this effect might indicate an improvement of the cerebellar inhibition [43]. Additionally, 4-AP has been reported to exert a robust neuroprotective effect on apoptotic cerebellar granule cells [44]. Furthermore, 4-AP (3 mg/kg, i.v.) has been shown to increase blood flow to various brain regions, being particularly significant in the cerebellum [45]. Thus, our results regarding the effects of 4-AP on cerebellar metabolism might be related to some of the above-mentioned actions of 4-AP. 

### 3.3. Systemic 4-AP Administration Does Not Alter Hippocampal Integrity 

4-AP (5 mg/kg, i.p.) administration had no detrimental effects on hippocampal neuronal integrity and survival as measured 3 days after the insult (Figure 3). This result agrees with a previous study where we showed that 4-AP i.p. at a dose of 3 mg/kg did not induce signs of neurodegeneration or neuronal death [20].

Many studies support the neuroprotective effects of K_v_ channel blockade [26,46]. For example, 4-AP (5 mg/kg, i.p.) abolishes the kainate-induced hippocampal neuronal cell death, as measured 3 days after kainate injection [47]. Both in vivo and in vitro stroke models have shown that potassium channel blockade attenuates ischemia-induced neuronal death and apoptosis [25]. Likewise, studies in cell cultures have reported that 4-AP prevents cerebellar granule neuronal cell death and apoptosis induced by low K^+^ (5 mM) serum-free conditions [44].

### 3.4. Effects of Systemic 4-AP Administration on Neuroinflammation Measured 3 Days After the Insult

Gliosis is a histopathological feature of epilepsy, including microglia and astrocyte activation [48,49,50,51]. Furthermore, complex and reciprocal regulatory interactions between microglia and astrocytes exist, and the temporal order of events is still unclear [52,53]. Nonetheless, it seems that the inflammatory effects of microglia may precede those of astrocytes [54,55,56].

Herein we show that systemic 4-AP administration did not result in microglia-mediated neuroinflammation analyzed by [^3^H]PK11195 autoradiography (Figure 4). By contrast, intrahippocampal 4-AP injection induced microglia-mediated neuroinflammation measured by in vitro [^18^F]GE180 autoradiography [21]. In other models of microglia activation, 4-AP has been shown to inhibit K^+^ outward current in rats [57] and to lessen the microglial production of neurotoxins and the consequent neuronal damage [58,59]. Thus, again it seems that despite being able to induce seizures, the effects of 4-AP, when administered systemically, clearly differ from those observed after intracerebral administration.

Astrocytes have been shown to be key players in the etiology and pathogenesis of epilepsy [49,51] and 4-AP has also been reported to non-selectively block K_v_ in astrocytes [24]. In our current study, astrocyte reactivity was observed 3 days after i.p. 4-AP-induced seizures. This result agrees with our previously reported data obtained in rats (4-AP, 3 mg/kg, i.p.) [20] as well as with data reported in mice (4-AP, 5.6 mg/kg, i.p.) [60]. Likewise, pre-treatment with 4-AP in rodent C8D1A-cultured astroglial cells increases LPS-stimulated astrocyte reactivity and production of pro-inflammatory cytokines [61].

Even though astrocyte reactivity is commonly associated with neurodegeneration, in our study, as depicted in Figure 5, we found astrocyte reactivity in the absence of hippocampal damage. Herein, it is interesting to notice that 4-AP directly acts on both neurons and astrocytes, and that astrocytes critically modulate the effects of 4-AP on neuronal integrity and survival [62]. Our result suggests that the astrocyte reactivity could be a consequence of the direct action of 4-AP on astrocytes and not as a response to neuronal damage. At this point, we cannot exclude the possibility that the astrocyte reactivity observed 3 days after the insult might in turn result in longer-term changes in hippocampal integrity. 

## 4. Materials and Methods

### 4.1. Animals

Thirteen adult Sprague-Dawley male rats (11–12 weeks old; Charles River, Sant Cugat del Vallès, Barcelona, Spain) were housed in pairs in standard cages on a ventilated rack (Tecniplast, Buguggiate, Italy). The animal room was under controlled conditions of temperature (22 ± 2 °C) and was under a light/dark cycle of 12 h (8 am–8 pm). Rats were left undisturbed for a minimum of 5 days to allow adaptation to the new housing environment. For environmental enrichment purposes, rats were provided with rodent chew sticks and plastic tunnels. Animals had free access to food and water. Access to food only was restricted for the 12 h before the PET scan procedures to ensure low blood glucose plasma concentrations for minimizing any eventual interference with the radiotracer tissue uptake. The body weights (BW) of the rats corresponding to the first PET/CT scan (basal) were 370.8 ± 8.7 g. Seven days later, corresponding to the second measurement, their mean BW was of 417.5 ± 11.4 g. All procedures were conducted following the animal welfare regulations of the European Union (2010/63/UE) and Spain (RD53/2013). The present study is part of a research project whose procedures were approved by the Animal Research Ethical Committee of the Complutense University of Madrid and by the Autonomous Community of Madrid (PROEX 222.1/20; 16 July 2020). All efforts were made to minimize the suffering of the animals.

### 4.2. Experimental Design

Longitudinal brain glucose metabolism activity was evaluated by [^18^F]FDG PET. By obtaining longitudinal measurements within the same animal, we could reduce the number of animals needed without putting at risk statistical power. Thus, on experimental day 1, we evaluated the basal metabolic activity (BASAL group), and on experimental day 7, the acute metabolic effects of 4-AP (Merck; 5 mg/kg, i.p., in 0.9% NaCl at a volume of 1 mL/kg) in the same animals (4-AP group) were evaluated 1 min after [^18^F]FDG injection.

**Figure 6:**
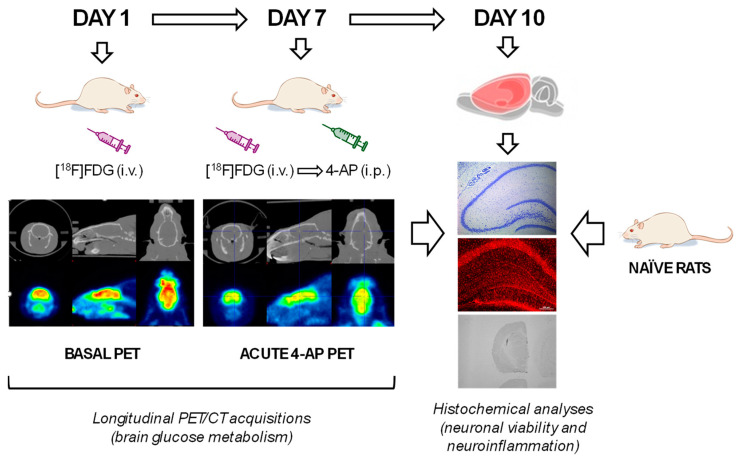
Schematic representation depicting the experimental design and procedures.

In parallel, a group of rats was kept naïve throughout the whole experiment to serve as a control group for histological studies (NAÏVE group; n = 6). All animals were sacrificed on experimental day 10. The experimental design is schematized in Figure 6.

### 4.3. [^18^F]FDG PET/CT Imaging

Brain glucose metabolism was evaluated by in vivo [^18^F]FDG PET imaging as previously reported [21,63,64]. Briefly, the rats were fasted overnight before the procedure to minimize the eventual competition between circulating glucose and [^18^F]FDG (the mean value for blood glucose concentration was of 102 ± 6 mg/dL). For basal metabolic activity evaluation (day 1), [^18^F]FDG was injected into the tail vein (13.05 ± 0.05 MBq −350 µCi- in approximately 0.2 mL of 0.9% NaCl; Curium Pharma, Madrid, Spain). On experimental day 7, the acute metabolic effects of 4-AP in the same animals (4-AP group) were evaluated by administering 4-AP (Merck; 5 mg/kg, i.p. in 0.9% NaCl at a volume of 1 mL/kg) 1 min after [^18^F]FDG injection.

Immediately after the injections, the rats were returned to their cages for a 30-min period to ensure the brain uptake of the radiotracer. After this uptake period, rats were anesthetized by inhalation of isoflurane/oxygen and placed on the bed of the tomograph. Image tomographic acquisitions were performed using a small animal dual PET/CT scanner (Albira PET/CT dual scanner, Bruker NMI, Karlsruhe, Germany). The acquisitions consisted of a 20-min static PET scan followed by a high-resolution computed tomography (CT) scan.

In both cases (day 1 and day 7), once the acquisitions were finished, PET images were reconstructed by using a maximum likelihood expectation maximization algorithm, applying decay, random, and scatter corrections.

### 4.4. PET Image Analysis by VOIs Analysis

The CT image of the skull of each rat was manually co-registered to a magnetic resonance imaging (MRI) rat brain template that contains the pre-defined brain regions to obtain a fitted spatial mathematical transformation. For each animal, the later CT transformation was applied to the corresponding PET image, allowing for the correct matching between the PET image and the MRI template as previously described [65]. Then, this co-registered PET image was overlaid onto the MRI template, allowing for the quantification of the tracer regional uptake values (kBq/mL; PMOD 4.1, PMOD Technologies Ltd., Zurich, Switzerland). In order to consider the individual body weights (BW), the specific [^18^F]FDG injected doses, and the corrected [^18^F]FDG uptake decay to the injection time, the regional glucose metabolic activities were normalized to standardized uptake values (SUV). Thus, SUV was calculated by applying the following equation: [Tracer uptake (kBq/mL) × BW (g)/Tracer dose administered (kBq)].

### 4.5. PET Evaluation by Statistical Parametric Mapping (SPM)

In experimental PET imaging studies, the regions that eventually show effects are often unknown in advance, and they might not correspond to the predefined anatomical VOIs. Thus, different from the traditional quantification using VOIs (see Section 4.4), SPM includes the whole brain in the analysis, without any preconception about the structures involved in the experimental paradigm [66]. This approach might unveil differences in subregions that are not detected by the VOI analysis. Additionally, when the regional uptake is normalized to the whole brain, it allows for comparing relative regional changes without interference from changes in blood brain flow and tracer peripheral uptake.

SPM comparisons were performed using MATLAB 9.7 (R2019b) software (The MathWorks, Natick, MA, USA) and SPM12 (Wellcome Trust Center for Neuroimaging, UCL, London, UK). To evaluate the effects of acute 4-AP injection, differences between BASAL and 4-AP-treated rats were calculated by paired *t*-test, setting a significance level threshold of 0.05 (uncorrected for multiple comparisons). The minimum cluster size of voxels was set to 100. The t-value threshold was set to show voxels with a correspondent *p* < 0.05. Parametric t-maps resulting from the comparison between the basal and the 4-AP groups were loaded in PMOD and fused to a rat brain T2-MRI template.

### 4.6. Brain Tissue Collection and Processing for Neurohistochemical Assessments

All rats were sacrificed by decapitation on experimental day 10 (3 days after 4-AP-induced seizures). Brains were collected, cut longitudinally into two halves, and stored at −80°C. Twenty-five µm thickness coronal slices at −20 °C were obtained using a cryostat (Leica CM1850, Leica Biosystems, Nubloch, Germany). Sections containing the dorsal hippocampus (Bregma −3.20 mm to −4.00 mm approximately) according to Paxinos and Watson [67] were thaw-mounted onto Superfrost Plus slides (Thermo Fisher Scientific, Dreieich, Germany), dried on a hot plate, and stored in slide boxes at −80 °C until the day of the assays.

### 4.7. Nissl Staining

Toluidine blue staining was used to qualitatively evaluate neuronal viability and hippocampal integrity. Thus, slides were stained in 0.5% toluidine blue for 2 min, decolorized, and dehydrated in graded ethanol: (i) 96% ethanol (10 s) and (ii) 100% ethanol (2 × 10 s). Then, the sections were cleared twice (2 and 5 min) in Neo-Clear^TM^ xylene substitute (Merck-Sigma-Aldrich, Darmstadt, Germany). Finally, the slides were mounted with DPX mounting medium (Herter, Barcelona, Spain). The dorsal hippocampus images were captured with a color digital camera (Leica DFC425, Leica Biosystems, Nubloch, Germany) coupled to a microscope (Leica DM 2000 LED, Leica, Biosystems, Nubloch, Germany) using Leica Application Suite (LAS) version 4.7. 

### 4.8. Fluoro-Jade C Labeling

To evaluate hippocampal neurodegeneration, Fluoro-Jade C staining was performed following the protocol previously described [68]. Thus, the brain sections were immersed for 2 min in 0.0001% Fluoro-Jade C (Millipore, Darmstadt, Germany) in PBS. Then, the slides were air-dried and cover-slipped with DPX mounting medium (Herter, Barcelona, Spain). The images were captured with a digital camera (Leica DFC3000G) coupled to a microscope (Leica DM 2000 LED) by using the FITC (fluorescein isothiocyanate) filter.

### 4.9. Microglia-Mediated Neuroinflammation by [^3^H]PK11195 Autoradiography 

As a marker of microglia-mediated neuroinflammation, we evaluated the translocator protein (TSPO; 18 kDa) by [^3^H]PK11195 autoradiography following the protocol previously reported [69] with minor modifications. The slides were dried on a hot plate at 37 °C (10 min), preincubated with 50 mM Tris-HCl pH 7.4 at RT (15 min), and then incubated with 1 nM [^3^H]PK11195 (PerkinElmer, Rodgau, Germany) in preincubation buffer. After 60 min incubating, the samples were washed in an ice cold preincubation buffer (2 × 5 min) and dipped in ice-cold distilled water. The slides were air-dried and exposed to Kodak BioMax MR autoradiography film (Carestream, Rochester, NY, USA) for approximately 2 months. After manual development, the film was placed onto a light box (Kaiser Prolite 5000, Kaiser Fototechnik, Buchen, Germany), and the images were captured with a camera (Leica DFC425) coupled to a stereomicroscope (Leica MZ6). Both delimitation and quantification steps were performed with ImageJ software version 1.54 g (NIH, available on the following website: https://imagej.net/ij/, accessed on 30 October 2023). The optical densities (O.D.) of the selected brain regions were used as an index of neuroinflammation degree. 

### 4.10. Reactive Astrogliosis 

As a marker of reactive astrogliosis, glial fibrillary acidic protein (GFAP) immunofluorescence was performed as previously reported [21,64,70]. Briefly, the slices were fixed in 4% formaldehyde, washed, and then blocked and permeabilized with 3% BSA and 0.1% triton X-100 in TBS (60 min). The slides were incubated overnight with the anti-GFAP-Cy3 antibody (1:500, Sigma Aldrich) in 1% BSA in TBS at 4 °C. Afterwards, they were washed in 0.1% Tween 20 dissolved in TBS (3 × 5 min) and cover-slipped with Mowiol mounting medium. The images were captured and evaluated using the same optical systems used for Fluoro-Jade C, but in this case using the TRITC (tetramethyl rhodamine iso-thiocyanate) filter. For each brain section containing the dorsal hippocampus, the fluorescence intensity was measured using ImageJ 1.54 g software. The results were expressed as a percentage vs. the NAÏVE group.

### 4.11. Statistics

Data are shown as mean ± SEM. The longitudinal data corresponding to within-individual differences in brain glucose metabolism between BASAL and 4-AP-treated groups were evaluated using a paired *t*-test. The data corresponding to the neurohistochemical studies was compared between the NAÏVE and 4-AP groups by unpaired *t*-test. The analyses were performed using SigmaPlot 11.0 (Systat Software Inc., San Jose, CA, USA). Statistical significance was considered when *p* < 0.05. Data are shown as mean ± standard error of the mean (ESM).

## 5. Conclusions

Summing up, even though 4-AP is relatively selective for A-type K^+^ channels at low concentrations, the selectivity is lost when used at higher doses. Thus, despite that 4-AP acts as a convulsant agent independently of the route of administration, acute generalized brain glucose hypometabolism and lack of neurodegeneration in response to 4-AP i.p. is clearly in opposition to the acute local hypermetabolism and extensive neuronal damage reported when 4-AP is administered intracerebrally. Therefore, it is evident that the route of administration and the dose have a significant impact on the 4-AP concentrations that reach the brain and, according to its potential beneficial or deleterious consequences. In addition, our study further points towards the cerebellum being more resilient to 4-AP-induced hypometabolism.

## 6. Future Perspectives

Regarding the PET neuroimaging data, it is appropriate to mention the limitations imposed by the spatial resolution, especially in rodent brains, the variability subjected to the researcher/operator criteria when delimitating VOIs, as well as the method used for data processing. All these factors may significantly impact the results obtained and their interpretation. Furthermore, subtle changes that might be of biological relevance might go unnoticed. Over the past decade, radiomics has become a field of high interest, being considered as a tool that, using automatized algorithms (artificial intelligence and machine learning), allows for the obtention of reliable quantitative data from images to further optimize diagnostics and to select proper pharmacotherapeutic strategies [71]. Its future application in preclinical settings would be a significant step ahead in the context of translational research [72,73].

## Data Availability

Data are available upon reasonable request to the corresponding author.

## Abbreviations

[^18^F]FDG	2-deoxy-2-[^18^F]Fluoro-D-Glucose
4-AP	4-aminopyridine
AMPA	α-amino-3-hydroxy-5-methyl-4-isoxazolepropionic acid
BW	Body Weight
CT	Computed Tomography
FITC	Fluorescein Isothiocyanate
GABA	Gamma-aminobutyric Acid
GFAP	Glial Fibrillary Acidic Protein
MRI	Magnetic Resonance Imaging
NMDA	N-methyl-D-aspartate
O.D.	Optical Density
PET	Positron Emission Tomography
SEM	Standard Error of the Mean
SPM	Statistical Parametric Mapping
SUV	Standardized Uptake Value
TRITC	Tetramethyl Rhodamine Iso-Thiocyanate
TSPO	18 kDa Translocator Protein
VOI	Volume of Interest
